# Systematic review and meta-analysis of the spatio-temporal changes in apparent tsetse fly density in Uganda from 1980 to 2022

**DOI:** 10.21203/rs.3.rs-7491610/v1

**Published:** 2025-09-25

**Authors:** Karla Rascón-García, Auther Tamale Wasswa, Beatriz Martínez-López, Giuliano Cecchi, Albert Mugenyi, Enock Matovu, Dennis Muhanguzi

**Affiliations:** University of California, Davis; Makerere University; University of California, Davis; Food and Agriculture Organization of the United Nations; Ministry of Agriculture, Animal Industry and Fisheries; Makerere University; Makerere University

## Abstract

Tsetse-transmitted trypanosomiasis continues to constrain more than 10 million km^2^ of high agricultural and livestock farming potential in sub-Saharan Africa (SSA). Despite its constraints to animal health and production, no accurate national and sub-national level data describing the distribution of tsetse flies has been produced for Uganda. To inform the tsetse fly density (flies/trap/day, or FTD) in Uganda and help advance along the progressive control pathway (PCP) for animal trypanosomiasis, we estimated FTD as an incidence rate across sub-counties in Uganda and explored factors influencing variations in FTD across studies. Tsetse fly publications (n = 2,288) were retrieved for Uganda from five life sciences databases, focusing on studies which inform the spatial distribution of tsetse flies, particularly reporting FTD across the nation. Following PRISMA guidelines, we conducted a systematic review and meta-analysis of 42 publications with the aim of producing an updated map to describe the spatial distribution of tsetse flies from pooled publication data. Current findings highlight substantial spatial data gaps with highly fragmented temporal collection periods. Of the 42 papers evaluated, only 20 reported FTD and were included in meta-analyses. To exhaust insights from extracted data, a zero-inflated multilevel Poisson was built to estimate the spatial distribution of tsetse flies. Meta-regression results found that the interaction of space (i.e. sub-county), time (i.e. collection period) and *Glossina* species explained the majority (95.93%) of observed FTD variations. Across all records obtained, the western region of Uganda was severely under-sampled. Despite the limitations, like underrepresentation of some regions and declining report of FTD over years, we identified a basis for future research, which should target identifying environmental and climactic predictors of tsetse fly habitats, and we established a solid foundation for the future development of a national-level information system for the vectors of trypanosomiasis (i.e. an ‘atlas’).

## Introduction

Tsetse transmitted African Animal Trypanosomiasis (AAT) is endemic across 38 countries in sub-Saharan Africa (SSA) and it constrains more than 10 million km^2^ of high agricultural and livestock farming potential in SSA^1,2^. Exacerbating global health conditions, tsetse flies are also responsible for transmitting human African trypanosomiasis (HAT), which is geographically restricted to areas (acute HAT in the east and chronic HAT in the northwest) known as ‘foci’^3,4^ in Uganda. AAT, on the other hand, is more fragmented and under-characterized in distribution^5^ and stagnates the economic progress and development of endemic countries like Uganda^6^. In Uganda, tsetse flies (*Glossina* sp.) infest an extensive area of approximately 140,000 Km^2^ of valuable land mass^7^, out of the 241,038 Km^2^ total landmass. This predisposes over 8 million cattle to AAT^8^ and 10 million of the worldwide 70 million people at risk of HAT were estimated to reside in Uganda^9^. The reported cases of HAT in Uganda have drastically reduced to near zero cases in the recent past^10^. Conversely, AAT control lags behind^11^. A relatively high national prevalence of AAT in cattle (22.15%), sheep (8.51%) and goats (13.88%) has been estimated using molecular techniques in 48 of 146 districts in Uganda^5^. Therefore, to progressively mitigate the burden of AAT, but also to contribute to HAT elimination^12^, tsetse control programs need to be scaled up and sustained.

Control and monitoring of tsetse population is an essential and integral part of planning, implementation and evaluation of vector control programs against AAT and HAT^12,13^. Tsetse abundance is highly dependent on numerous ecological factors such as temperature, humidity, and habitat moisture^14^. Other factor such as pathogen presence, vegetation cover, *Glossina sp*. and physiology additionally influence tsetse abundance^15^. Apparent tsetse fly density expressed as flies per trap per day (FTD) is the most used entomological indicator for assessing vector abundance in a specific geographic location^16^. However, the estimation of FTD is influenced by factors such as type of trap used, seasonal changes, duration of trapping and *Glossina sp*. The FTD provides knowledge on the risk of transmission of both AAT and HAT in a particular geographic region^17^.

Several studies have been conducted in different areas in Uganda to determine the apparent tsetse density and tsetse species distribution^17–22^, and in a recent continental review^23^ for 1990–2020, FAO identified over 40 papers allowing to map the reported occurrence of tsetse flies in Uganda. These studies ranged from entomological surveys, *Glossina* sp. distribution prediction and tsetse-based longitudinal interventional studies employing baited and non-baited traps, tiny targets and insecticide treated cattle or restricted insecticide application protocols (RAP). Furthermore, a considerable amount of entomological data has been generated across the country through existing surveillance systems, and oftentimes this valuable information remains dispersed and fragmented among the archives of stakeholders like the Ministry of Agriculture Animal Industry and Fisheries (MAAIF). As such, there is need to put together existing data, both published and unpublished, on tsetse apparent densities in Uganda using systematic approaches.

This study was aimed at informing the spatial distribution of tsetse flies across the nation by pooling data from published literature across 1980–2022. More specifically, we conducted systematic review and meta-analysis on the apparent tsetse density by using both published and unpublished sources and providing previously unavailable estimates of pooled tsetse density across Ugandan districts and sub-counties. Additionally, a consolidated overview of the spatial distribution of tsetse abundance in the different administrative units of Uganda has been developed. This information will contribute towards more effective development of vector control interventions, ultimately contributing to advancing Uganda along the progressive control pathway (PCP)^13^ for AAT and the 2030 World Health Organization’s (WHO) goal of complete interruption of HAT transmission^10^. Also importantly, the database associated with this meta-analysis and that associated with our previously published meta-analysis of AAT^9^ will be used as a strong basis to develop a national tsetse and AAT atlas for Uganda^24^, following the example of several other countries such as Ethiopia^25^ and Kenya^26^.

## Methods

### Search Strategy and Application Programming Interfaces (APIs)

**Figure 1:**
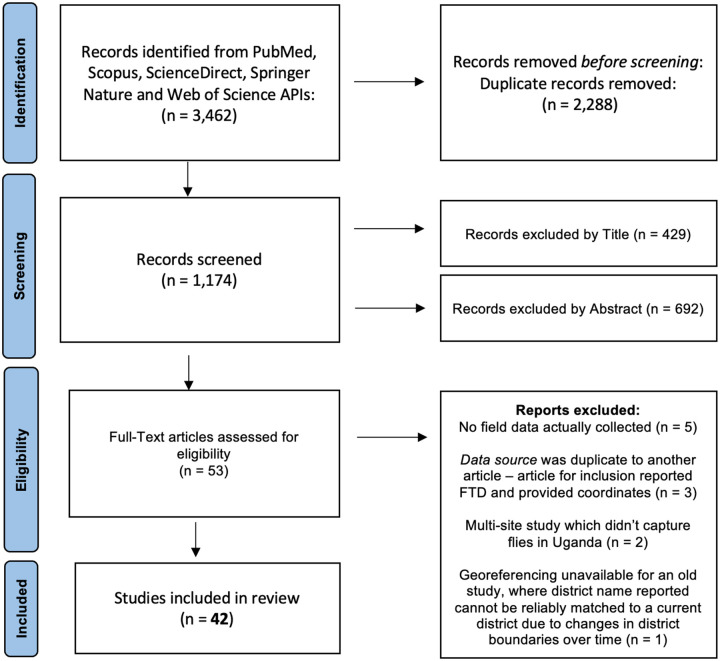
Flowchart depicting article selection and the inclusion/exclusion process

This study was conducted in accordance with the Preferred Reporting Items for Systematic Reviews and Meta-Analyses (PRISMA) guidelines^27^. Articles published on PubMed, Scopus, ScienceDirect, Springer Nature and Web of Science databases were searched and retrieved through each database’s application programming interface (API) – API requests were submitted January 4, 2023. Batch retrieval of peer-reviewed article information was by use of standard query language implementation. Search terms included the following: “trypanosomiasis AND Uganda”, “tsetse trypanosomiasis AND Uganda”, and “glossina AND Uganda.” APIs were accessed through Python modules: PyMed^28^ and Elsapy^29^ for PubMed, Scopus, and Science Direct databases. Springer Nature and Web of Science APIs were accessed through direct URL requests^30,31^. Following the automated retrieval of records, the first author of this paper worked independently screening articles, conducting full-text evaluations, and extracting data from the included articles in accordance with the PRISMA flow described in the Fig. 1. A PRISMA checklist is provided in Supplementary Information.

### Inclusion/Exclusion Criteria

The articles included in this study had to be full-text available, report tsetse fly collections in Uganda, be written in English, and published between 1980–2022. This study was not limited to publications which reported tsetse fly density (flies/trap/day, or FTD) but rather included all records reporting the presence of tsetse flies. If an intervention method (e.g., randomized control trial, case-control, etc.) was involved, only baseline data or control arm data were included to estimate FTD in the associated study area. Finally, articles were excluded from FTD estimation if they involved experimental studies or were laboratory-based.

### Data Extraction and Geocoding

For each article included in this review, the following attributes were collected: the reported district, publication year, date and year of tsetse fly collection, whether or not available records from the publication describe trap-specific information, the trap type (e.g., biconical, pyramidal, etc.), georeferencing coordinates (Latitude/Longitude, Easting/Northing), trap specific flies per trap per day, total tsetse flies captured, and *Glossina* species if speciation was performed. If trypanosome infections were investigated or if baiting was used, these attributes were recorded.

For records where coordinates were not reported or available, we utilized Google’s Geocoding API^32^ to obtain latitude and longitude coordinates for each village provided. District and sub-county were assigned to as attributes to all coordinates based on 2020 definitions. This allowed us to visualize sub-county and district-level summaries of all data collected.

For articles where the tsetse fly collection year was not reported, data were assumed to have been collected the same year as the publication year. Moreover, to produce the most comprehensive database from pooled literature, total tsetse fly captures were calculated, where possible, from the provided FTD estimates according to the number of traps and trapping hours (e.g., 48-hours, 72-hours, etc.) reported. Finally, corresponding authors to six publications were contacted where either coordinates or FTD estimates were referenced but not directly available from their associated publications; this way, the most complete database was compiled from published literature.

### Publication Bias

Publication bias was assessed by both 1) visual examination of a funnel-plot and 2) the Luis Furuya-Kanamori (LFK) index^33^. LFK values more extreme than ±2 indicate the presence of publication bias as indicated by “major asymmetry” in the observed funnel plot. In the event of a non-symmetrical funnel plot, imputed effect size estimates were derived using the trim-and-fill method^34^ to evaluate the effect of possible publication bias.

### Data Analysis

The analytic methods in this study were determined by the different data types available across publications. The 42 articles collected can be categorized into four, basing on different outcome formats. These categories were composed of studies that had:

1. Explicitly reported FTD estimates (*k* = 20)
2. Reported total tsetse fly captures and the number of traps used, but might have failed to report observation days (*k* = 16)
3. Focused on studying the *Glossina* genome yet still provided coordinates for where flies were captured (*k* = 16)
4. Reported large tsetse fly captures but neither reported FTD nor the number of traps used (*k* = 4)

A formal meta-analysis was conducted on the 20 articles (Category I above) which reported clear FTD estimates. Category II data were evaluated under a zero-inflated multilevel Poisson model. Noteworthy is that Category I and II data above had 14 publications in common; two separate models were built to describe apparent tsetse fly density from two available definitions (i.e., flies per trap and flies per trap per day). The latter two (Categories III and IV) were not interrogated under any inferential approach. These data were only retained to descriptively produce a point distribution map informed by all 42 articles.

### Point Distribution Map - Spatial Reporting of Tsetse Flies in Uganda

All 42 publications provided information on tsetse captures at some capacity. Independent of total flies captured, we produced a map to describe the general spatial distribution of tsetse fly capture reports using the provided study site or geocoded coordinates. A point map was produced to describe the species distribution of captured tsetse fly species across all 42 publications retained. All maps were produced using R version 4.3.1, with the primary packages used including *sf*^35^ and *ggplot2*^36^.

### Meta-Analysis: Tsetse Fly Incidence Rate (Flies/Trap/Day)

For articles which reported FTD (*k* = 20), a meta-analysis of incidence rates was implemented. More specifically, we evaluated the number of events (total tsetse captures reported) per trap-days reported to obtain FTD estimates. Between-study variance (heterogeneity) was calculated using the inverse variance index statistic (I^2^ statistic)^37^. Heterogeneity was estimated by a random-effects model using a Freeman-Tukey Double arcsine transformation for estimating incidence rates^38^ and significance assessed using the Cochran Q test^39^.

Forest plots were generated to displaying an FTD estimate for the entire nation. Sub-group analyses were conducted by region (Central, Eastern, Northern, and Western), *Glossina* species reported, and data collection period (1980–2000, 2001–2005, 2006–2010, 2011–2015, and > 2016). To make use of the high-resolution data available (i.e., latitude and longitude coordinates) all maps produced informed estimates for a sub-county map according to 2020 boundary definitions. For spatial mapping of tsetse FTD, Jenks natural breaks were used to create maps with categories of three classes.

### Meta-Regression

Univariate and multivariate meta-regression analyses were conducted to investigate sources of heterogeneity in our meta-analysis, focusing on district, sub-county, tsetse fly collection period, and *Glossina* species reported as potential sources. Explanatory variables with a p-value < 0.25 in univariate analyses were included in multivariate meta-regressions to ascertain the amount of heterogeneity explained by multiple variables. All analyses were conducted using R version 4.3.1, with the primary packages used including *meta*^40^ and *metasen*s^41^.

### Zero-Inflated Multilevel Poisson Regression

Sixteen publications reported a count of total tsetse fly captures and the number of traps used but did not specify the observations hours associated with the observed captures (e.g., 48-hours, 72-hours). To model the incidence of tsetse flies per trap among these records, we tested for zero-inflation by comparing the number of predicted to observed reports of zero captures. Zero-inflation was confirmed and selected over a conventional multilevel Poisson model. To estimate tsetse fly counts per trap, we included 1) a random intercept for each latitude/longitude pair, which represented individual traps, and 2) a random intercept for each publication. Both district and sub-county maps describing model estimates were produced to visualize results using quartile breaks (0.25, 0.50, and 0.75) to create categories of four classes.

## Results

### Literature Search

A total of 3,462 records were retrieved across all five databases under the pre-defined search query terms. Across all databases, 2,288 duplicate records were identified resulting in 1,174 publications available for Title and Abstract screening. After inspection of titles and abstracts, 53 publications were determined to be eligible for full-text review. Upon full-text evaluation, 42 articles were retained as records which met inclusion criteria and were eligible to meaningfully contribute to our research aim, which was to describe the spatial distribution of tsetse fly vectors across Uganda (Fig. 1). Among these 42 articles evaluated, 68 districts were represented (see Supplementary Table S1 for article attributes). Moreover, 4,013 records (e.g., peer-reviewed publications, gray literature, etc.) were extracted across all publications.

**Figure 2:**
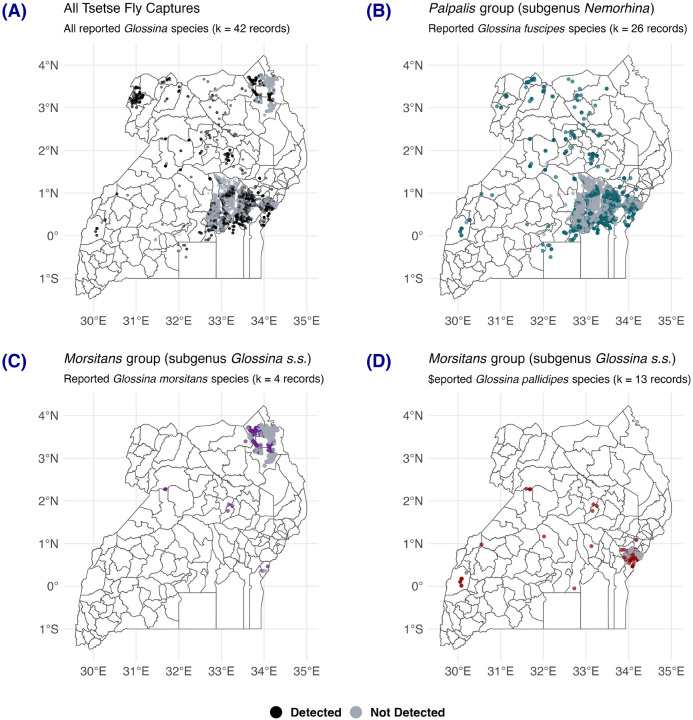
Map of tsetse fly detection reports by *Glossina* species. (A) all *Glossina* species across all 42 publications obtained (*n* = 4,013 records), (B) *Glossina fuscipes* (C) *Glossina morsitans* and (D) *Glossina pallidipes*.

Thirty-three publications (78.6%) performed species identification of captured tsetse flies. However, the total records from these publications only made up 864 of the 4,013 (21.5%) records compiled. All remaining records informed tsetse fly captures but did not report the species (Supplementary Figure S1). A map describing the spatial distribution of *Glossina* species was produced (Fig. 2).

Most records came from the eastern region of the country, just north of Lake Victoria. Some studies reported the presence of multiple *Glossina* species, but the dearth of taxonomical reports at the species level limited our ability to define accurately the spatial distribution of *Glossina* species.

### Meta-Analysis of Tsetse Fly Density (Flies/Trap/Day, or FTD)

#### FTD by Study Attributes

Across the 20 articles included in the meta-analysis, 39,169 total tsetse flies were captured. Among these studies, most of the traps used were Biconical^42^ (81.0%), followed by Pyramidal^43^ (18.7%) and less than 1% reported Monoscreen^44^ traps. Only 44 districts (~ 32% of 136 districts) were sampled within these 20 articles, highlighting a significant under sampling of districts across Uganda (Table 1).

**Figure 3:**
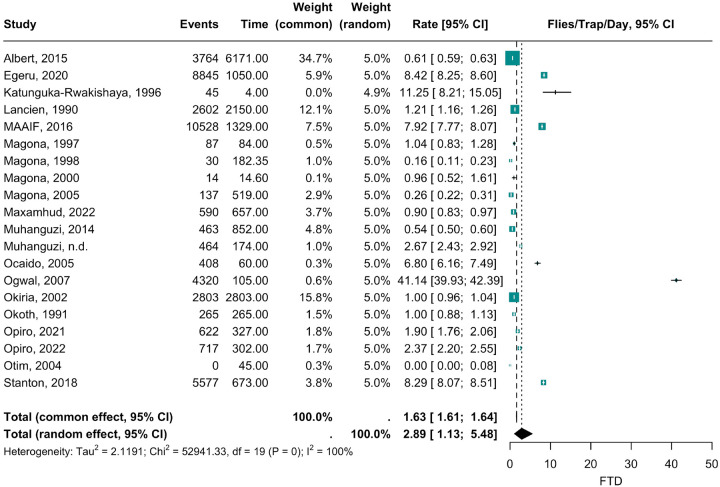
Forest plot of tsetse fly density (flies/trap/day) meta-analysis results

A forest plot describing FTD estimates across the 20 publications analyzed is provided in Fig. 3 below. Most publications estimated an FTD between 0–12 except for Ogwal, 2007^45^ which estimated a high FTD of 41.14 FTD.

#### Publication Bias

A funnel plot assessing publication bias is provided in Supplementary Fig. S2. Moderate asymmetry (2.21 LFK score) was observed. Consequently, imputed effect size estimates were obtained using the trim-and-fill method^34^. Forest and funnel plot results provided in Supplementary Fig. S3 and S4, respectively. The FTD across all 44 districts was estimated at 1.76 under a trim-and-fill, a considerably lower density estimate compared to 2.89 FTD from the original 20 publications (Fig. 3). FTD estimates from the imputed trim-and-fill method are not reported as primary results and not visualized on a map as funnel plot asymmetry, in this study’s context, was believed to more likely to be explained by alternative factors, like heterogeneity in sampled land use areas.

#### FTD by District and Sub-County

**Figure 4:**
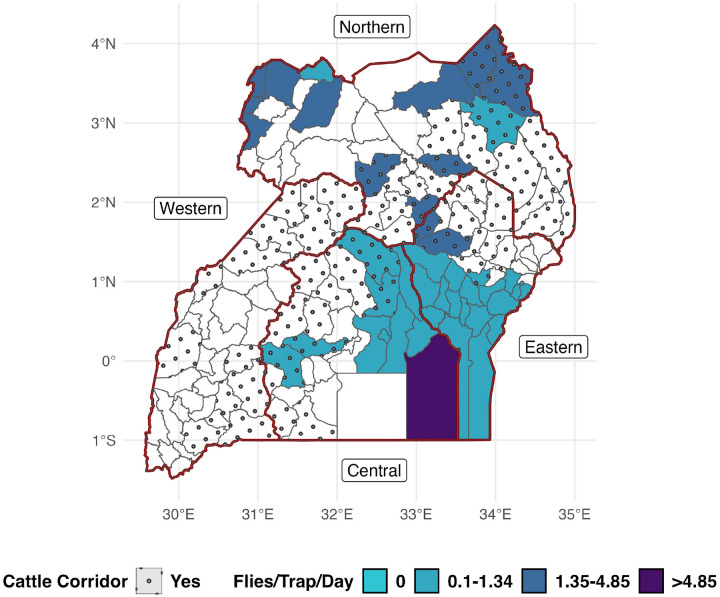
Tsetse flies/trap/day estimates by District, all Glossina species. Uses Jenks natural breaks obtained from district-level estimates for class definitions. No district nor sub-county estimated an FTD of zero. Subnational boundaries (i.e., regions) are denoted by thick red borders. The cattle corridor denotes districts with cattle density of > 50 head / square km^46^.

Natural breaks using the Jenks algorithm with three classes were used to produce the map in Fig. 4. FTD estimates were visualized at the district level (Fig. 4) to describe estimates at the actional MAAIF level as well as the sub-county level (Supplementary Fig. S5) to more accurately describe the spatial distribution of tsetse flies.

Forty-three districts were accounted for with only 13 districts (~ 22.4% of 58 districts) from the Cattle Corridor sampled at least once (Fig. 4). The most sampled region, both in terms of the greatest proportion of districts sampled and the greatest number of traps reported, was the Eastern Region. FTD estimates by sub-county suggest a persistent or endemic presence of tsetse flies with no district nor sub-county estimating an FTD of zero. Small, higher density tsetse fly foci (best seen in Supplementary Fig. S5) were suggested.

Tsetse fly distribution maps stratified by collection period and *Glossina* species are provided in Supplementary Figures S6 and S7, respectively.

#### Meta-Regression analyses

Region, district, sub-county, collection period, and *Glossina* species were all significant predictors of FTD in univariate meta-regression models (p < 0.25) with Glossina species influencing heterogeneity the most (20.39% of variation), see Table 2.

The additive effect of sub-County and *Glossina* species describe a considerable amount of FTD variation (56.45%) while the interaction of former two with collection period explained the overwhelming majority of observed FTD variations (87.32%).

#### Zero-Inflated Multilevel Poisson Regression – Tsetse Flies per Trap

**Figure 5:**
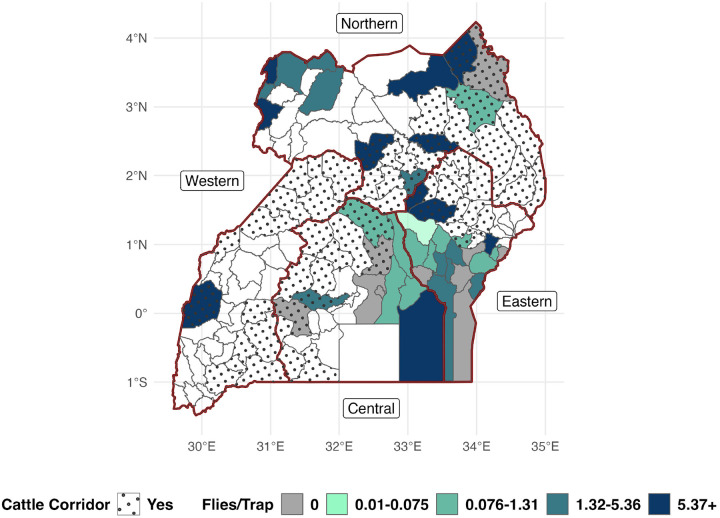
Zero-Inflated model estimates by District all *Glossina* species; uses quartile breaks for class definitions. Regions are denoted by thick, red borders. The cattle corridor denotes districts with cattle density of > 50 head / square km^46^.

Sixteen publications reported tsetse fly captures but did not report observations days, preventing us from being able to include these records in a formal meta-analysis. We included a random intercept for each latitude/longitude pair (representing individual traps), and a random intercept for each publication. No additional covariates were considered in these models. Zero-inflation was confirmed among these data and tsetse fly incidence rates (flies per trap) were estimated and visualized at the district level (see Fig. 5).

Forty-three districts (31.6%) were accounted for among sampled districts. Density estimates suggest dispersed tsetse fly foci across the nation, possibly associated with tsetse suitable habitats across topographically distinct regions of the nation. A map describing sub-county estimates is provided in Supplementary Figure S8.

## Discussion

In this study we compiled published literature from 1980 through 2022 with the aim of describing the spatial distribution of tsetse flies in Uganda. A total of 42 records were obtained, which informed the spatial presence of *Glossina* species in the nation. Of these, 20 articles reported apparent tsetse fly density as defined by number of flies captured per trap per day, or FTD, on which a meta-analysis was conducted. Forty-three of all districts (31.6%) were represented. Incidence rate meta-analysis results found considerable gaps in spatial sampling with no district from the Western region included. Underrepresentation of western region districts could be because most surveys were biased towards sleeping sickness which has never been reported in Western Uganda. Additionally, south-western region commercial farmers extensively use acaricides/insecticides some of which (cypermethrin, deltamethrin and organophosphates) are tsetse-effective making researchers believe that the tsetse (AAT) problem there was not so significant.

The average FTD estimate across these districts was 2.89, with no district nor sub-county sampled estimating an FTD of zero. When investigating variables which inform most of the observed variability in apparent density, *Glossina* species, sub-county, and collection period informed the greatest proportion (R^2^ = 87.32) of observed variation. However, we suspect this high R-squared is likely an artifact of the underlying data. Most tsetse fly captures came from 2006–2010 (see Supplementary Figure S9), and approximately half (51.08%) of captured tsetse flies were not distinguished to species level. Despite this, these results corroborate the notion that tsetse flies still transmit *Trypanosoma sp* and remain a significant problem across the 42 districts of Uganda: calling for sustained control of tsetse transmitted AAT^5^.

A paralleled analysis of publications (*k* = 16), which did not consider observation days but looked at the incidence of tsetse flies per trap, was conducted using a zero-inflated multilevel Poisson regression. Estimates described tsetse fly populations to be dispersed in spatial clusters.

Results from both the meta-analysis and zero-inflated model suggest tsetse flies to have established a persistent presence in the sampled areas. This indicates that such spatial clusters (sub-counties) should be targeted when implementing risk-based tsetse control programs^13^.

Both inferential models found the Western region of Uganda to be considerably under-sampled and should be a targeted region in future tsetse fly research and monitoring events. The Eastern region of Uganda, on the other hand, has received strong investigative support with at least 40.9% of records associated with this region. This investigative bias was associated with acute sleeping sickness programs like stamp-out sleeping sickness which were geared towards eliminating acute sleeping sickness from the south-eastern Uganda as a public health problem by the year 2020^47^. The factors influencing the observed disproportionate spatial sampling (i.e., minimal studies focused on the Western region) were not directly explored here, but possible explanations could the encouraged HAT research in the Eastern region or an underlying assumption that acaricide use in the West might suggest a low-risk tsetse fly challenge in the West. Despite some limitations, this study remains of high relevance especially to the key players in tsetse and AAT control. Like all meta-analyses, this study carried the strength of having pooled data sources across multiple databases and spanning over 30 + years. By pooling all publications that informed the presence of tsetse flies in different formats, we were able to explore the apparent density of tsetse flies through different angles. We believe that the greatest contribution of this work is having highlighted the spatial gaps in tsetse fly sampling (Supplementary Figure S10), inconsistent FTD reporting, and infrequent performance of tsetse fly identification. Additionally, this meta-analysis exercise confirms that tsetse remains abundant and spatially clustered across 42 districts of Uganda, indicating risk-based tsetse control programs need to target these tsetse clusters.

Several limitations were encountered and each, however, provides the research community with opportunities to improve. The first limitation included the inconsistency of FTD reporting. FTD estimates were, at times, summarized over a study region and not always available at the trap-level. Whether FTD is a primary objective of a research study or not, we strongly advise the research community to collect and, ideally, make freely available these descriptive estimates for public use or at least collect data from which these estimates can be calculated^24^. In so doing, our collective efforts can allow us to shed more reliable insights on the spatial distribution of tsetse flies, which is an indispensable foundation for the control of Trypanosomiasis.

In addition to consistent FTD reporting, species identification was not performed half of the time (51.08%), greatly limiting our ability to describe tsetse fly species distribution in Uganda. Additionally, though we produced maps describing district-level estimates, all sub-county maps reveal the limitation of district-level estimates in that in many cases district estimates were derived from very small samples. District estimates (whether high- or low-density estimates) should be weighted with explicit consideration of the number of study sites associated with them.

## Conclusions

Estimates described tsetse fly populations in Uganda to be dispersed in spatial clusters with inferential models suggesting tsetse flies have established a persistent presence over time and space in sampled areas. Meta-regression results suggest that sub-county, *Glossina* species, and collection period inform the greatest proportion of observed heterogeneity. This implies that ecological and seasonal factors are likely to inform the spatial distribution of *Glossina* species, which should be interrogated in future research.

Combined with a previous meta-analysis of on AAT^5^, the present study also represents a first major step towards the development of a national-level information system on animal trypanosomiasis and its vectors in Uganda (i.e. an ‘atlas’), which is considered a key requirement for the country to advance along the PCP.

## Supplementary Material

This is a list of supplementary files associated with this preprint. Click to download.

- tsetsesupplementaryfiles.docx

## Figures and Tables

**Table 1:** Tsetse Flies/Trap/Day by Study Attributes (k = 20 articles)

Attribute	No. Studies	Total Tsetse Fly Captures	Trap-Days Contributed	Estimated F/T/D	95% CI	I^2^	*p*-value
**Collection Period** *							
<2000	8	5846	5548	1.0537	(1.03, 1.08)	98.96	< 0.001
2001–2005	2	545	579	0.9413	(0.86, 1.02)	99.89	< 0.001
2006–2010	3	13661	6949	1.9659	(1.93, 1.99)	99.99	< 0.001
2011–2015	2	1085	1179	0.9203	(0.87, 0.98)	99.74	< 0.001
>2016	5	21144	3512	6.0205	(5.94, 6.10)	99.95	< 0.001
**Glossina Species** *							
*Glossina* (Non-Specific)	7	9497	3011	3.1541	(3.09, 3.22)	99.91	< 0.001
*Glossina fuscipes*	12	12916	6898	1.72	(1.84, 1.91)	99.92	< 0.001
*Glossina pallidipes*	3	89	268	0.3321	(0.27, 0.41)	87.69	< 0.001
*Glossina morsitans*	2	19373	723	26.7953	(26.42, 27.17)	99.27	< 0.001
*(Multiple) Glossina fuscipes & pallidipes*	1	406	201	2.0199	(1.83, 2.23)	-	1.00
*No Flies Captured*	10	0	6666	0.0000	(0.00, 0.0006)	0.00	0.8346
**Region** *							
Central	5	5965	3246	1.8376	(1.79, 1.88)	99.97	< 0.001
Eastern	12	9363	10144	0.9230	(0.90, 0.94)	99.48	< 0.001
Northern	7	26953	4377	6.1579	(6.08, 6.23)	99.94	<0.001
Western	-	-	-	-	-	-	-

* Some studies contributed information to multiple regions, multiple time periods, and multiple Glossina spp across records

**Table 2:** Univariate and Multivariate meta-regression analysis results

Model	Variable	p-value	R^2^
Univariate	Region	<0.0001	8.15
	District	<0.0001	18.58
	Sub-County	0.0878	6.70
	Collection Period	0.0677	1.09
	Glossina Spp	<0.0001	20.46
Multivariate	District + Collection Period	<0.0001	21.94
	District + Glossina Spp	<0.0001	32.97
	Collection Period + Glossina Spp	<0.0001	19.96
	Sub-County + Collection Period	0.0004	15.59
	Sub-County + Glossina Spp	<0.0001	56.45
	District * Collection Period	<0.0001	23.02
	District * Glossina Spp	<0.0001	33.73
	Collection Period * Glossina Spp	<0.0001	19.45
	Sub-County * Collection Period	0.0410	8.90
	Sub-County * Glossina Spp	<0.0001	32.83
	District + Collection Period + Glossina Spp	<0.0001	41.02
	District * Collection Period * Glossina Spp	<0.0001	41.66
	Sub-County + Collection Period + Glossina Spp	<0.0001	87.32
	Sub-County * Collection Period * Glossina Spp	<0.0001	41.66

Note: an asterisk (*) denotes an interaction between the two neighboring variables, while a plus symbol (+) denotes an additive effect.

p-values in bold indicate models with a p-value < 0.05

## Data Availability

The dataset analyzed in this study is available upon reasonable request from the corresponding author. Detailed descriptive statistics are provided in Supplementary Files.
